# “Identity theft” in *BRCA1/2*: impact of positive genetic test results and risk-reducing interventions

**DOI:** 10.3389/fgene.2024.1380637

**Published:** 2024-07-09

**Authors:** Jonathan M. Adler, Sharlene Hesse-Biber, Memnun Seven, Andrew A. Dwyer

**Affiliations:** ^1^ Department of Psychology, Olin College of Engineering, Needham, MA, United States; ^2^ Department of Sociology, Boston College, Chestnut Hill, MA, United States; ^3^ Elaine Marieb College of Nursing, University of Massachusetts Amherst, Amherst, MA, United States; ^4^ William F. Connell School of Nursing, Boston College, Chestnut Hill, MA, United States; ^5^ P50 Massachusetts General Hospital–Harvard Center for Reproductive Medicine, Boston, MA, United States

**Keywords:** breast cancer, coping, genetic testing, hereditary cancer, narrative identity, identity theft

## Abstract

Individuals harboring breast cancer gene 1/2 (*BRCA1/2*) pathogenic variants are at increased lifetime risk for developing cancer. Learning one’s *BRCA1/2* carrier status is a watershed moment that can result in psychological distress, anxiety, and depression, as well as feelings of vulnerability and stigma. However, emotional and coping responses to learning one’s *BRCA1/2* carrier status and after risk-reducing interventions (i.e., preventative bilateral mastectomy) are variable, and existing literature reveals mixed and sometimes contradictory results. Drawing on the concept of narrative identity from the field of psychology, we sought to examine if “identity theft” (the sudden overtaking of one’s narrative agency by an external force) may help explain the heterogeneity of emotional and coping responses following the revelation of *BRCA* carrier status and the subsequent medical intervention one may receive. This Perspective explores *BRCA* related identity theft using two case studies. Narrative analysis of qualitative interviews uncover the ways that patients experience the disintegration (theft) of their identity as well as their efforts to build and reintegrate a new *BRCA* carrier identity. This initial qualitative exploration provides preliminary support for the relevance of narrative identity and identity theft to hereditary cancer. We posit that applying the lens of identity theft may hold promise as a unifying concept, integrating across the variable emotional and coping responses among *BRCA* carriers. Employing a lens of identity theft may help inform the development of tailored narrative interventions as part of precision healthcare to support active coping and psychosocial wellbeing.

## 1 Introduction

Genetic testing for pathogenic variants in breast cancer gene 1/2 (*BRCA1/2*) can identify individuals at increased cancer risk (specifically breast and ovarian), provide opportunities for risk management (i.e., cancer surveillance) and cancer prevention (i.e., risk-reducing surgery or medication), and benefit blood relatives via cascade carrier screening (The National Comprehensive Cancer Network, 2023). Systematic reviews show that despite many potential physical and psychological benefits (i.e., access to risk management to improve personal and familial health, testing-informed targeted treatment approaches), testing positive for *BRCA1/2* may also be associated with psychological distress, anxiety, depression, and diminished quality of life (Lombardi et al., 2019; Butler et al., 2021; Isselhard et al., 2023). Individuals may also often experience vulnerability, stigma, guilt, and decreased self-confidence (Mella et al., 2017; Hesse-Biber et al., 2022). Moreover, feelings of loss frequently accompany learning one’s *BRCA1/2* carrier status as intimate relationships, reproductive decisions, and self-concept can be altered (Rapport et al., 2018; Hesse-Biber et al., 2022). It is worth noting that the negative impact is not universal, and some individuals may only experience short-term distress following the revelation of *BRCA1/2* carrier status and cancer risk management, cancer prevention, and cancer treatment(s). Indeed, the literature reveals disparate emotional and coping responses to *BRCA1/2* variant status and treatment. Currently, it remains unclear if a unifying element could help explain the varied emotional responses of *BRCA* carriers that could inform tailored psychosocial support. Herein, we introduce “identity theft” as a new concept in relation to identity in individuals who harbor pathogenic *BRCA1/2* variants.

We build on the broad area of research on what psychologists have termed “narrative identity” (McAdams, 1996; McAdams, 2001; McAdams and McLean, 2013). Narrative identity is the internalized, evolving story of the self that integrates the reconstructed past, perceived present, and imagined future (McAdams, 1996). A robust body of scholarship examines how major life challenges impact people’s narrative identity across adulthood (Mitchell et al., 2021), identifying key narrative arcs that are disrupted (McLean et al., 2020). Disruptions include inserting contamination sequences, wherein stories that begin positively end negatively (Adler et al., 2008) with loss of agency, communion, and coherence (Adler et al., 2015; Adler et al., 2018). We posit that learning of one’s *BRCA1/2* carrier status may be illustrative for understanding identity theft–the loss of one’s narrative authority over one’s life story. Such a concept may hold broad explanatory potential, expanding our understanding of identity processes in the context of hereditary cancer (e.g., hereditary breast and ovarian cancer, HBOC) while opening new ways of conceptualizing identity disruption in response to life challenges.

Identity theft is a relatively new concept in the psychology literature. Work in narrative identity has informed tailored approaches to supporting individuals with acquired disability (Adler et al., 2021; Adler et al., 2022) and demonstrated a buffering effect on accelerated biologic aging associated with the stress of chronic caregiving (Mason et al., 2019). Currently, it is unclear how identity theft may relate to individuals with hereditary cancer risk. Investigating identity theft in cancer is vital for understanding the way(s) the phenomenon impacts health (i.e., risk management, intrafamilial communication of *BRCA* risk), coping responses, and pathways to improved psychosocial wellbeing. We explore identity theft in individuals with pathogenic *BRCA1/2* variants and uncover how patients experience identity theft and their efforts at rebuilding a new identity. We envision this initial exploration will spur additional research into the experience of cancer-related identity theft and inform the development of tailored narrative approaches to improve health outcomes as part of precision healthcare. In addition, we hope that deploying this psychological concept will augment sociological approaches to illness that contextualize individual experience in broader social and structural systems (Williams, 2000).

## 2 Methods

Two cases are presented using a qualitative, descriptive inquiry. Cases are drawn from a larger study that employed an explanatory sequential, mixed-methods approach to examine genomic healthcare utilization and intrafamilial communication of *BRCA* risk among ethnically and racially diverse individuals (Hesse-Biber et al., 2023). The Boston College IRB approved the study (protocol #16.109.01) and participants provided informed consent.

### 2.1 Participants

Two participants harboring pathogenic BRCA1/2 variants were selected based on shared demographic characteristics. Both cases offer distinct insights into the experience of identity theft in the context of hereditary cancer risk. Participant # 011 (pseudonym: Patricia) is a 35-year-old, married female who identifies as a Black woman. She holds an advanced degree with a middle-class income ($76–100k). Genetic testing at age 22 revealed a *BRCA2* variant. Participant #003 (Kendra) is a 28-year-old, married, and self-identified Black woman from a mixed-race background (Caucasian, Black, Hispanic). She holds an Associate’s degree with an upper-middle class income ($101–125k). She tested positive for a *BRCA1* variant at age 21 years. Developmentally, both participants are in early mid-life, a period when issues of identity, intimacy, and generativity converge in psychological salience while people navigate career, relationship, and parenting decisions (Wilt et al., 2010). Prior research has identified narrative differences in people with genetic vulnerability at different stages of the life course (Hamilton et al., 2016).

### 2.2 Procedures and analysis

In-depth semi-structured interviews were conducted (SH-B) and transcribed verbatim (#011:04/27/2022, 73 min, ∼20,000 words; #003: 4/14/2022, 111 min, ∼16,000 words). Three investigators (SH-B, MS, AAD) conducted qualitative analysis on transcriptions as previously described (Hesse-Biber et al., 2023). Two cases were purposefully selected from the larger sample as potential illustrations of identity theft. An independent investigator (JMA) with expertise in the study of narrative identity independently conducted a narrative analysis of the two transcripts to describe identity theft experiences. A narrative phenomenological approach (Moustakas, 1994) was utilized to describe the nature and form of experiences as filtered through the subjective narration of those experiencing it (Priest, 2002). This narrative phenomenological approach begins with a deductive identification of a phenomenon of interest (identity theft in this study,) and then seeks to zero in on the parts of participants’ narratives that focus on that phenomenon. Then, inductive methods are used to explore participants’ narration of the phenomenon, prioritizing their first-person perspectives. In this study, the investigator closely read the transcripts, noting key quotes regarding the experience of identity theft, the phenomenon of interest. He then reread both transcripts multiple times, seeking to richly capture the participants’ narration of their experiences of different identity thefts (one focused on the revelation of *BRCA* + status, one focused on medical trauma associated with risk-reducing surgery), while putting the two cases in dialogue with each other. All investigators discussed the cases and narrative analysis to triangulate the findings, then synthesized case descriptions.

### 2.3 Positionality

Investigators are focused on the interface between illness and identity and coping responses in genomic healthcare. Their scholarly work is informed by their professional experiences and personal and/or friends/family members experience with cancer. The cases are Black cisgender women, and investigators include two cisgender White men and two White cisgender women (including one first-generation immigrant woman). Differences in racial/gender identity between participants and investigators introduce the likelihood of some slippage in interpretation. No scholarly commitment can completely overcome the vital differences in lived experience between participants and scholars. The investigators are committed to addressing and dismantling cancer disparities and believe that amplifying the voices of people of color in research is essential.

## 3 Results

### 3.1 Patricia’s story

Patricia was 17 years old when her life got “*turned upside down*” when her parents returned from a medical appointment and announced that Patricia’s mother was going to die. Patricia said that her mother’s physician *“told my Dad, “your wife has stage four metastatic breast cancer; she has 3 months to live,” and he walked off*.*”* The brusque revelation sent their family into crisis. The news of her mother’s impending death was traumatic and had a significant emotional impact on Patricia. She stopped dating, and daily family life revolved around being together as a family before her mother died several months later.

Five years later, Patricia pursued genetic testing for hereditary cancer risk while trying to focus on life as a 22-year-old college graduate navigating her entry into the field of public health. When she received the results, she felt unprepared to discover she had a pathogenic *BRCA2* variant. *“I was in denial. I told the lady at the front desk, I said, “y’all got the wrong person.” And I just walked out.”* Patricia could not incorporate her own genetic risk into her identity and withdrew from medical care for several months.

There was not a specific incident that made her return to seek care. She shared, *“I was not myself,”* indicating the revelation that her *BRCA2* carrier status had stolen something from her identity. Furthermore, after months of feeling lost, she decided to get *“fully educated on myself*.*”* This phrasing–*“educated on myself”*–suggests that learning her *BRCA*2 carrier status substantially disrupted her identity and she needed to find someone to “teach” her about her new self. In both of these comments, Patricia verbally distinguishes her prior self from her self following test results.

In searching for the “right” physician, she found it important to find another Black woman—like herself. She had encountered racism in the medical system and, working in public health, she was deeply attuned to structural inequities that Black people face in healthcare. She described her new physician as *“aggressive, but in a good way”* adding, *“I think what really helped was my doctor was a Black woman. She had my stress*.*”* In describing their first encounter, Patricia repeatedly used the word *“surrender*.*”* It was as though her doctor facilitated Patricia’s ultimate concession that the *BRCA2* variant was in charge now, and she had to attend to its demands. Patricia said, *“She was very aggressive, and I was very defensive, very resistant* […] *what she said to me that really helped me to surrender in a sense. I told her to stop. I told her to just leave me alone, because she was treating me like I had cancer. And she told me, “No, you don’t have cancer.” She said, “but you’re at a higher risk for cancer.” She says, “and I have to be aggressive, because you have options. Your mom didn’t have options. Other patients, some of them don’t have options.” And she says I need you to know and explore your options. And that’s the time when I just threw my hands up and surrendered. And I was just like, okay, okay.”*

Her physician established a connection by recognizing that Patricia was not herself. *“I’m glad she was the way she was. Because that’s what I needed … She kind of snapped me out of it, like “wake up,” you know, out of this state, this thought process that I was not myself … she just knew that I was not myself.”* Patricia could not recapture her former identity, yet her physician helped jolt Patricia out of a defensive fog into a new reality. *“After I kind of surrendered, so to speak to, you know, the situation, I begin to then feel educated and equipped to be able to manage my health going forward.”*

Patricia began monitoring her body, and keeping regular preventative care appointments, yet she did not communicate her genetic risk with anyone. *“I felt I was grieving and processing this all for myself.”* Over time, she began developing a new sense of self. Six months after discovering her *BRCA2* carrier status, she started dating again, and met the man she would ultimately marry. She felt supported by him when disclosing her *BRCA2* carrier status and envisioned a long-term future with him. Patricia’s physician encouraged her to consider risk-reducing surgery. With a new normal and a supportive partner, she felt ready to undergo a preventative double mastectomy and speak with her family about hereditary cancer risk. *“I didn’t really share with them until almost 10 years later when I had my double mastectomy. Over 10* *years, I was fully educated and equipped to be able to share this news*.*”*

After recovering from her preventative bilateral mastectomy, she and her partner were married. About 6 months later, Patricia became pregnant. *“We really hadn’t planned on it, but that’s when I also started to educate myself on like, you know, passing this mutation on to my children and what that means, and so felt very confident that, if my children did inherit my mutation, that there was something we could do, you know, that there was a game plan. And so it didn’t alter our life or change our decision. It just, you know, made us more well-informed*.*”* Unlike the dramatic destabilization following her mother’s cancer diagnosis and her own *BRCA2* carrier status, Patricia fully embodied a new identity, enabling her to look toward the future with hope and confidence.

### 3.2 Kendra’s story

Kendra also experienced identity theft as the result of a *BRCA* variant, yet it was not the revelation of the variant itself that stole her identity, but rather the trauma she experienced following medical intervention. Kendra had a significant history of cancer on both sides of her family. At age 21, when Kendra’s paternal aunt received her second breast cancer diagnosis, Kendra and her younger sister opted for genetic testing, revealing they both had pathogenic *BRCA1* variants. Rather than feeling devastated, the results confirmed her feeling that *BRCA* would be part of her future, *“I felt it in my bones that I was positive … It was like, this has been with me my whole life, and I know it’s there. It just doesn’t have a name yet*.*”*

Her father felt tremendous guilt for passing the *BRCA1* variant to his daughters. *“My Dad, he still won’t talk about it to this day. When I had decided to get a double mastectomy, he did not talk to me about it.”* Being a *BRCA1* carrier was a source of bonding between Kendra, her younger sister, and aunt noting, *“within the family it’s just kind of a heavy thing that exists between the different generations.”* In this case, *BRCA1* was an inter-generational presence in the family, a story that was shared by some and silenced by others (Werner-Lin and Gardner, 2009), with power to divide and unite members.

Kendra was married and wanted children by age 25. Knowing her *BRCA1* carrier status drove her to be proactive. *“I wanted to be free of that weight. I wanted to be free to be healed if I had children. I wanted it to be the past.”* After medical consultation, she opted for a preventative double mastectomy, a decision she met with clarity. However, she did not fully understand how profoundly the decision would impact her identity. *“Before that appointment, I believed I was going to be the same as I was then [pre-surgery]… and even now, like, after I’ve had the double mastectomy, I’m not. I’m not the same at all.”* While Kendra expected changes to her body and sense of femininity, she did not anticipate what would happen after surgery. The procedure (preventative mastectomy and placement of tissue expanders) was uneventful, yet days after hospital discharge she developed an infection and felt like one breast would *“pop”*. Kendra said, *“Luckily there was like a BRCA breast cancer Facebook group and I submitted a photo of my breast to this basically asking, like, “has anyone else had this? Is this normal?”And a woman on the page said you need to go to the emergency room.”* Kendra’s expander had developed an infection. She recalled *“I’m sweating, I’m hot, I’m cold, I’m starting to get delirious. I was starting to have delirious visions of places, and things, and people in my life. And they immediately took me into surgery. My infectious disease doctor told me I was 2 days away from dying.”* The surgeons removed one expander, and she was hospitalized for 2 weeks. She recalled lying in the hospital bed, *“It was so weird to be able to touch my chest, my ribs from that area, because you almost go, “What is it like?” Like experimenting on yourself, you go, well, what is this like now?”* Without fully realizing it, she had experienced identity theft—a rupture in her sense of self so severe that she would ultimately need to find a new way of being. Kendra described the magnitude of this theft in narrating what followed.

After returning home, a subsequent infection required a week-long hospitalization. Physicians recommended removing her other tissue expander following a planned 6-month recovery. The subsequent surgery and recovery were largely uneventful, she felt permanently altered by the experience. *“I was fiercely independent … And so, to be taken to such a dependent level, that was very difficult … I was strong. I was so strong, you know … and I lost that, you know, through it all … You lose your sense of femininity, and then you lose, like, these other parts of you too, that are just gone. Some are just completely gone, and I don’t think I will ever fully get back.”* In addition to losing her sense of strength and control, Kendra experienced a spiritual crisis and questioned her religious identity. She described a meeting with the congregation leader, *“He told me that I should trust God and not have a double mastectomy. I remember being so mad at this man, and I said, “It has killed probably hundreds of women in my family!”…In the religion, women are not supposed to be opinionated. And I was very opinionated.”* She summarized, *“My double mastectomy was the reason why I left the religion.”*

Following the trauma of her hospitalizations and her loss of identity, the couple was poised to pursue their deferred (now altered) dream of having children. *“We’re going to get a surrogate because I don’t think I could handle the pelvic exams. We’re going to do IVF, and then they’re going to genetically test the eggs to see if they have the genes, and then we’re going to pick out the ones that don’t … I acknowledge the fact that it’s vital to ensure that this doesn’t happen, but, it’s such a … I just wish I could have kids the normal way.”* Kendra is still working on navigating her life following her traumatic *BRCA*-related experiences.

For Kendra, the discovery that she had the *BRCA* mutation was not a devastating revelation. Rather, it was more like a confirmation of something she had unconsciously anticipated. However, the unexpected medical-related traumas she experienced in proactively responding to her genetic heritage forever altered her identity, robbing her of her sense of strength and independence, her religion, and her plans to get pregnant.

## 4 Discussion

These two stories unfold in different ways, highlighting the varied experiences of identity theft. For Patricia, the *BRCA*2 variant became the narrator of her life story, forcing her to abandon her “old” self and surrender to new *BRCA* life experiences. In contt, the *BRCA*1 variant did not act as the ‘thief’ of Kendra’s identity. Rather, the consequences of risk-reducing surgery overtook her identity, disrupting her self-concept, religious beliefs, and path to parenthood. These cases reveal identity theft as a new and insightful concept for understanding how identity is fundamentally affected by having a pathogenic *BRCA1/2* variant.

In this manuscript, portray one description of how having a pathogenic variant in *BRCA1/2* can impact identity. We focus on identity because it is a salient site of disruption for patients and identity may also be a unifying variable for understanding the heterogeneous psychosocial impacts of hereditary cancer diagnosis and treatment reported in the literature. Indeed, data show that cancer-affected, *BRCA* + individuals have increased psychological distress, anxiety, and depression compared to counterparts without a cancer diagnosis (Ringwald et al., 2016). Yet for *BRCA* + people without a cancer diagnosis, study results on distress, depression, and quality of life are mixed (Isselhard et al., 2023). Similarly, predictors of emotional and coping responses are divergent and sometimes contradictory. A 2021 review on body image in BRCA + individuals post-mastectomy found mixed results across studies spanning diminished, unchanged, and improved body image post-surgery (Torrisi, 2021). Importantly, protective factors shape adaptation to *BRCA* + status, including coherent self-concept, positive body image, and strong social networks supporting psychological adaptation and coping (Lombardi et al., 2019; Butler et al., 2020; Torrisi, 2021; Hesse-Biber et al., 2022). Meta-analysis shows that optimism and coping buffer against breast cancer-related distress, anxiety, and depression (Fasano et al., 2020). However, parsing the heterogeneity of psychosocial effects has been challenging to date. Identity may therefore offer a focus for future investigations aimed at explicating the psychosocial ramifications of hereditary illness experience.

It merits noting that a key aspect of Patricia’s case was the death of her mother shortly after being diagnosed with breast cancer. A recent systematic review concluded that children often hide their grief from others to psychologically protect themselves and others around them (Wray et al., 2022). Moreover, grief is a highly individual response. Some people may prefer support from peers or family in a one-to-one context or a group setting, while others may need specialized support from healthcare professionals. Regardless, social network support often dwindles rapidly. In the context of a parental death from cancer (like Patricia’s story), waning support is a critical factor for coping and adaptation. A 2021 study of 622 young adults found that acute grief experiences and reactions in the first 6 months following death of a parent were associated with long-term grief resolution 6–9 years following loss (Bylund-Grenklo et al., 2021). Furthermore, family narratives of loss in the context of hereditary cancers shape risk communication and prevention actions not only via their content, but also via their tone and framing (Campbell-Salome and Rauscher, 2020). Such findings are consistent with Patricia’s trauma related to her mother’s death as it took Patricia several years to adapt to her mother’s death, her own *BRCA2* variant status, and “reintegrate” her identity.

In Kendra’s case, her *BRCA1* findings had a relatively minimal effect on her possibly because she may have anticipated the positive result and/or that she underwent testing with her sister. Indeed, the literature supports that siblings who undergo testing together and have concordant results are more likely to cope better (Smith et al., 1999). Kendra’s identity theft is related to her experiences with risk-reducing interventions. A large 2020 multicenter study examining the quality of life in young breast cancer survivors following risk-reducing surgery (i.e., breast conserving, unilateral, bilateral mastectomy) found that measures of physical functioning, sexuality, and body image improved over time across surgical interventions (Rosenberg et al., 2020). However, young people who underwent bilateral mastectomy exhibited consistently worse scores on measures of sexuality and body image–findings that are reflected in Kendra’s narrative.

Applying the concept of narrative identity from psychology to the experiences of individuals with pathogenic variants in *BRCA1/2* offers a new perspective on identity processes in *BRCA*, one that augments sociological perspectives with a deep attention to the personal subjective narration of this experience. We posit that identity theft may serve as a possible unifying concept with explanatory potential for understanding variability in the emotional and coping responses of *BRCA1/2* carriers. A deeper, more nuanced understanding of coping response is critical for reaping the full potential of genomic healthcare. Indeed, coping response is a key driver of intrafamilial risk communication and cascade screening, one shaped by familial narratives of vulnerability (Werner-Lin and Gardner, 2009) and which may mitigate hereditary cancer risk in blood relatives (Sarki et al., 2022). A new conceptualization of identity disruption could inform more individualized, person-centered approaches to psychosocial support for *BRCA1/2* carriers that align with precision healthcare (National Research Council US Committee on A Framework for Developing a New Taxonomy of Disease, 2011). Thus, future precision healthcare may not only encompass individualized interventions for prevention, diagnosis, and treatment but also tailored narrative identity-based approaches.

### 4.1 Limitations

A relative strength of this transdisciplinary investigation is the rigorous analytic approach employed by experienced qualitative investigators across disciplines (psychology, sociology, and nursing). The cases were purposefully sampled from a larger set of interviews to explore the phenomenon of identity theft and not necessarily representative of the broader phenomenon. Our should be considered transferable rather than generalizable. The depth of our exploratory qualitative analyses were limited by the space requirements of a Perspectives manuscript and this report does not capture all nuances of the phenomenon of identity theft. We did not aim to definitively explain the heterogeneity of findings in the literature. Rather, we sought to introduce new concept (identity theft) as a potential new lens for understanding the range of emotional and coping responses and inform tailored approaches to psychological support aligning with precision healthcare.

### 4.2 Conclusion

Case studies provide initial support for a new conceptualization of identity and *BRCA.* Identity theft may be relevant for integrating variable emotional and coping responses among *BRCA1/2* carriers. The concept may help inform targeted, individualized interventions to support active coping and wellbeing. Future directions may include larger qualitative studies and developing narrative interventions as part of precision healthcare. Such investigations could also examine if personalized narrative approaches effectively address genomic healthcare disparities.

## Data Availability

The data presented in the study are deposited in the Harvard Dataverse repository, accession number https://doi.org/10.7910/DVN/WBZVEK.
